# Longitudinal data in peripheral blood confirm that PM20D1 is a quantitative trait locus (QTL) for Alzheimer’s disease and implicate its dynamic role in disease progression

**DOI:** 10.1186/s13148-020-00984-5

**Published:** 2020-12-09

**Authors:** Qi Wang, Yinghua Chen, Benjamin Readhead, Kewei Chen, Yi Su, Eric M. Reiman, Joel T. Dudley

**Affiliations:** 1 ASU-Banner Neurodegenerative Disease Research Center, Arizona State University, Tempe, AZ USA; 2 Banner Alzheimer’s Institute, Phoenix, AZ USA; 3 Icahn School of Medicine at Mount Sinai, New York, NY USA

**Keywords:** Alzheimer’s disease, Epigenetics, PM20D1, Mixed-effects model

## Abstract

**Background:**

While Alzheimer’s disease (AD) remains one of the most challenging diseases to tackle, genome-wide genetic/epigenetic studies reveal many disease-associated risk loci, which sheds new light onto disease heritability, provides novel insights to understand its underlying mechanism and potentially offers easily measurable biomarkers for early diagnosis and intervention.

**Methods:**

We analyzed whole-genome DNA methylation data collected from peripheral blood in a cohort (*n* = 649) from the Alzheimer’s Disease Neuroimaging Initiative (ADNI) and compared the DNA methylation level at baseline among participants diagnosed with AD (*n* = 87), mild cognitive impairment (MCI, *n* = 175) and normal controls (*n* = 162), to identify differentially methylated regions (DMRs). We also leveraged up to 4 years of longitudinal DNA methylation data, sampled at approximately 1 year intervals to model alterations in methylation levels at DMRs to delineate methylation changes associated with aging and disease progression, by linear mixed-effects (LME) modeling for the unchanged diagnosis groups (AD, MCI and control, respectively) and U-shape testing for those with changed diagnosis (converters).

**Results:**

When compared with controls, patients with MCI consistently displayed promoter hypomethylation at methylation QTL (mQTL) gene locus PM20D1. This promoter hypomethylation was even more prominent in patients with mild to moderate AD. This is in stark contrast with previously reported hypermethylation in hippocampal and frontal cortex brain tissues in patients with advanced-stage AD at this locus. From longitudinal data, we show that initial promoter hypomethylation of PM20D1 during MCI and early stage AD is reversed to eventual promoter hypermethylation in late stage AD, which helps to complete a fuller picture of methylation dynamics. We also confirm this observation in an independent cohort from the Religious Orders Study and Memory and Aging Project (ROSMAP) Study using DNA methylation and gene expression data from brain tissues as neuropathological staging (Braak score) advances.

**Conclusions:**

Our results confirm that PM20D1 is an mQTL in AD and demonstrate that it plays a dynamic role at different stages of the disease. Further in-depth study is thus warranted to fully decipher its role in the evolution of AD and potentially explore its utility as a blood-based biomarker for AD.

## Background

Over a decade of genetics research on Alzheimer’s disease (AD) has identified 30+ susceptibility genes which altogether account for less than 50% of the heritability of late-onset Alzheimer’s disease (LOAD) [1]. With continuous efforts to thoroughly characterize the genetic risk for LOAD, emerging evidence suggests that epigenetics also plays a significant role in disease pathogenesis, progression and resilience [2–4]. Among the various epigenetic modifications, DNA methylation is the most widely studied mechanism due to its interpretable relationship to disease-associated gene expression, the availability of different experimental assays and advanced analysis tools, thus enabling high throughput processing. It has also attracted increasing attention as a potential biomarker, with accumulating evidence indicating that abnormal methylation can be used for detection and diagnosis of disease, prediction of response to therapeutic interventions and prognosis of outcome [5]. For AD, the methylation profile from peripheral tissues such as blood would be especially useful as a diagnostic tool due to the noninvasive, easily measurable characteristics and the possibility of longitudinal study [6].

Specifically for LOAD, in postmortem brain tissues, cell type-specific methylation signatures and differential methylation dynamics were reported for several brain pathology-related genes such as ankyrin 1 (ANK1) [7, 8], histone deacetylases (HDACs) [9, 10] and homeobox genes (HOXs) [11, 12]. More recently, analysis from biologically and technically independent datasets focusing on the comparison between samples from healthy controls and patients with advanced-stage AD shows that only one gene, peptidase M20-domain-containing protein 1(PM20D1), a biosynthetic enzyme for a class of N-lipidated amino acids in vivo, consistently displayed promoter hypermethylation in patients with AD [13]. Furthermore, it has been demonstrated that PM20D1 is a methylation and expression QTL coupled to an AD-risk associated haplotype defined by rs708727 and rs960603, which displays enhancer-like characteristics and contacts the PM20D1 promoter via a haplotype-dependent, CCCTC-binding-factor-mediated chromatin loop [13]. Nevertheless, these findings, which were all observed in various brain tissues, are not necessarily translatable to the whole blood due to the highly dynamic nature of peripheral tissues and the heterogeneity of disease. Multiple studies have attempted to reproduce the observations of methylation changes in brain and peripheral blood, but few consistent results have emerged. For example, hypermethylation of ALK1 in two cortical regions (superior temporal gyrus and prefrontal cortex) of AD subjects has been observed; however, these alterations were not observed in whole blood obtained premortem from the same individuals [8]. TNF-α shows significant hypomethylation in the cortex samples of AD patients but not in their blood samples [14]. These findings indicate that some of the epigenetic mechanisms being uncovered in AD pathology are only relevant to brain cells, not blood cells. Furthermore, many epigenetic alterations observed in blood cells could not be detected in the brain [15]. A global correlation of epigenetic changes in the brain with peripheral tissues is yet to be established.

Still there is evidence indicating that blood DNA methylation dynamics may mediate detectable transcriptomic changes [8] and many DNA methylation variations have consistent effects across tissues [16]. Analysis of methylomic co-variation between tissues, specifically between whole blood and different regions of brain demonstrated that for a portion of the methylation sites, blood methylation levels are correlated with those from brain, and suggested the utility of using a blood-based approach to identify potential biomarkers of psychiatric disease phenotypes [17]. Given the difficulty in accessing and collecting brain tissue samples especially longitudinally to track disease diagnosis and progression, valuable information could still be obtained from blood-based DNA methylation studies [6].

We set out to utilize the data available from the ADNI study which comprised a large cohort with both blood-based longitudinal DNA methylation data and cross-sectional gene expression data, by starting from baseline DNA methylation measurements in the stable diagnostic groups to detect the differential methylated regions (DMRs) at the group level, then tracking the dynamic change of the DMR among the different diagnostic subgroups, including those converters. It is one of the ongoing efforts to exploit the data to seek understanding of the heterogenicity and dynamic nature of AD. The ample information possessed by the longitudinal data in the ADNI cohort could help validate the previous findings observed in brain tissues while providing unprecedented perspectives into the dynamics of the epigenetic background of AD. Based on the ADNI data, we now report the preliminary findings that are observed at PM20D1 locus: (1) confirmation of PM20D1 as a methylation and expression QTL coupled to an AD-risk associated genotype defined by rs708727 in peripheral blood, mostly consistent with what is been observed in the brain tissues from the hippocampus and the frontal cortex; and (2) the methylation profile’s moving direction at the early stage of AD, which is contrary to what is observed in brain tissues with advanced-stage AD (Braak staging ≥ 5) [13]. We modeled the alteration of methylation levels at the promoter regions of the gene as a function of disease progression and validated the findings in a separate cohort with both methylation and gene expression data from brain tissues. This work helps characterize a comprehensive picture for the epigenetic change at the PM20D1 gene locus associated with the onset, as well as progression of AD and suggests its potential as a blood-based biomarker.

## Results

### Methylation data processing and quality check (QC)

**Fig. 1:**
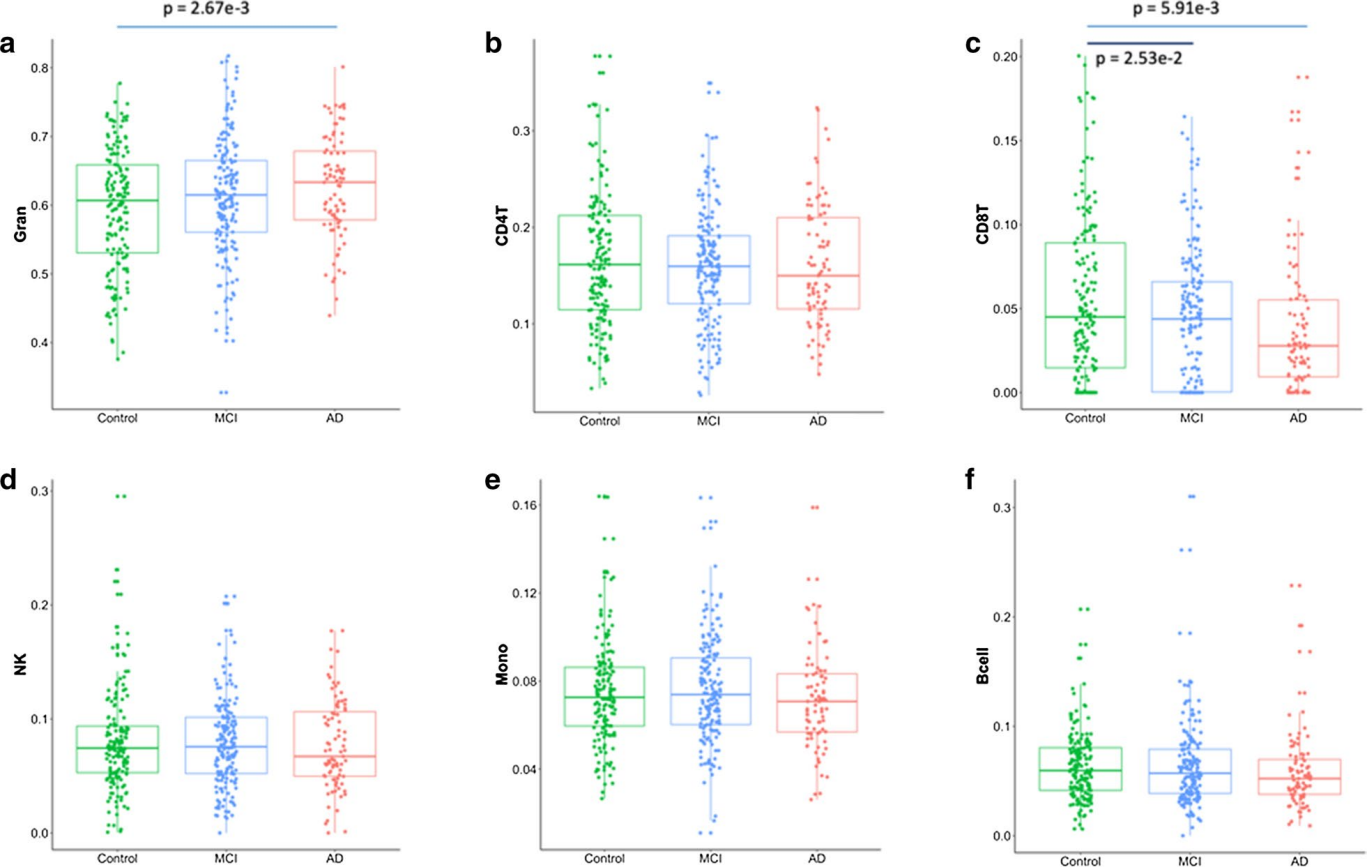
Comparison of individual cell type across control (*n* = 162), MCI (*n* = 175) and AD (*n* = 87) groups with stable diagnosis at baseline measurements. Shown in **a** granulocytes (Gran), in **b** CD4T cells, in **c** CD8T cells, in **d** natural killer (NK) cells, in **e** monocytes (Mono) and in **f** B cells. Blue line indicates comparison between AD cases versus control subjects; black line indicates comparison between MCI cases versus control subjects. *P* Value for the differences in cell composition estimates across groups as per *T* test is indicated

The details of the ADNI study, the experiment design for methylation array and initial data QC are available online at: http://adni.loni.usc.edu/methods/documents/ and http://adni.loni.usc.edu/data-samples/access-data/. Briefly, DNA was isolated and plated out at NCRAD and DNA methylation profiling was performed at AbbVie for a total of 1920 samples, including 1719 unique samples and 201 technical replicates (653 unique individuals). Longitudinal DNA samples at baseline, and +up to 4 years, sampled at ~ 1 year apart were obtained from all subjects. Samples were randomized using a modified incomplete balanced block design, whereby all samples from a subject were placed on the same chip, with remaining chip space occupied by age- and sex-matched samples. Subjects from different diagnosis groups were placed on the same chip to avoid confounding. Unused chip space was leveraged for technical reproducibility assessment via replicated DNA samples. Sample and probe quality control, including detection P values, checking the sex of samples and sample identity, removed 15 samples from the data. The released raw data from ADNI methylation array consist of 1905 samples at > 850,000 CpG sites, creating unique challenges in data processing and QC. We chose to process the data with the most recently developed ‘bigmelon’ package in R, a memory efficient method to import the raw data and export the data matrices without any filtering steps. All the downstream analyses were accomplished by the ChAMP pipeline for an integrated workflow, which employs a set of tools for filtering, normalization, batch correction and cell type correction for data from whole blood samples. Preprocessing of the data resulted in 685,446 probes for 1,905 samples. Singular value decomposition (SVD) analysis [18] after batch correction found no covariates contribute to significant components of variation. Mean value for each estimated cell proportion shows the cell type profile agrees well with known cell type profile for whole blood samples, with granulocytes/neutrophils making up 50–60% of the samples and lymphocytes/monocytes making up the rest (Fig. 1). At baseline, the proportion of granulocytes was higher (*p* = 0.0027, fdr = 0.048, two-sided Student’s *t* test), and that of CD8T cells was lower (*p* = 0.0059, fdr = 0.053, two-sided Student’s *t* test) in the AD patients, indicating an alteration of immune response in the AD patients. All other cell types remain comparable among the subjects. This observation also does not change in the full longitudinal data time frame.

### Differentially methylated regions (DMRs) at baseline

**Fig. 2:**
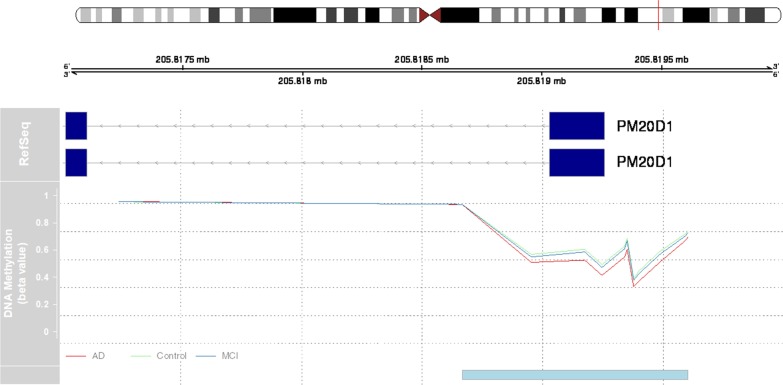
Graphical representation of differentially methylated region (DMR) near PM20D1 gene locus. Genomic location is indicated by chromosome position based on Genome Reference Consortium Human Build 37 (GRCh37). Transcripts are indicated by light blue arrows. Solid line represents *β* values for all the CpGs constituting the significant region, where AD is colored in red, MCI in blue and control in green

We first focused on those subjects (579 out of 649) whose age > 65 at the baseline measurements (LOAD). A subset of the subjects (424) kept a stable diagnosis during the whole sampling time frame throughout all the visits; thus, their baseline measurements were used for DMR detection. A region of ~ 900 bp on chromosome 1 demonstrated hypomethylation in the AD patients in comparison with control (*p* = 7.48E−06, fwer = 0.004), as well as AD vs MCI (*p* = 9.11E−05, fwer = 0.048). This is also the only DMR passing the corrected fwer cutoff (0.05). This region also demonstrated moderate hypomethylation when we compared MCI against control (*p* = 0.00237, fwer = 0.748), indicating a gradual methylation decrease during disease progression (Table 1, Fig. 2, Additional file 1: Table S1). Interestingly, this region lies within the promoter region of gene PM20D1, the recently reported mQTL/eQTL for AD, albeit it is hypermethylated in some brain regions of subjects with advanced LOAD [13]. We therefore focused on the longitudinal data for the 10 CpG probes from the Illumina EPIC array in this region (Table 2) to fully dissect the methylomic changes for PM20D1 in peripheral blood, throughout the course of disease progression.

**Table 1:** The differentially methylated regions (DMRs) in PM20D1 promoter region as detected by the comparisons among control, MCI and AD

Comparison	Rank	Chr	Start	End	Width	Value	Area	*p *value	fwer	*p*.value Area	Fwer area
AD versus control	1	chr1	205,818,668	205,819,609	941	−0.476	5.23	7.48E−06	0.004	0.000217	0.112
AD versus MCI	1	chr1	205,818,956	205,819,609	653	−0.373	3.73	9.11E−05	0.048	0.000615	0.276
MCI versus control	5	chr1	205,818,956	205,819,609	653	−0.143	1.43	0.00237	0.748	0.00597	0.952

**Table 2.** 10 CpG probes and their respective *β* values from EPIC array in the DMR region at PM20D1

CpG probes	Control	MCI	AD	MAPINFO	UCSC refgene group
cg17178900	0.568 ± 0.200	0.549 ± 0.193	0.509 ± 0.208	205818956	Body
cg14159672	0.605 ± 0.233	0.586 ± 0.225	0.526 ± 0.241	205819179	1st exon
cg14893161	0.493 ± 0.220	0.472 ± 0.217	0.414 ± 0.216	205819251	5′UTR; 1st exon
cg07533224	0.627 ± 0.221	0.609 ± 0.215	0.549 ± 0.232	205819345	TSS200
cg12898220	0.685 ± 0.207	0.667 ± 0.201	0.605 ± 0.224	205819356	TSS200
cg05841700	0.390 ± 0.190	0.378 ± 0.182	0.330 ± 0.177	205819383	TSS200
cg11965913	0.447 ± 0.240	0.427 ± 0.234	0.373 ± 0.229	205819406	TSS200
cg24503407	0.589 ± 0.213	0.568 ± 0.210	0.510 ± 0.220	205819492	TSS1500
cg16334093	0.720 ± 0.136	0.705 ± 0.210	0.672 ± 0.147	205819600	TSS1500
cg07157834	0.739 ± 0.127	0.727 ± 0.129	0.695 ± 0.136	205819609	TSS1500

### Allelic dosage effect in the DMR

**Fig. 3:**
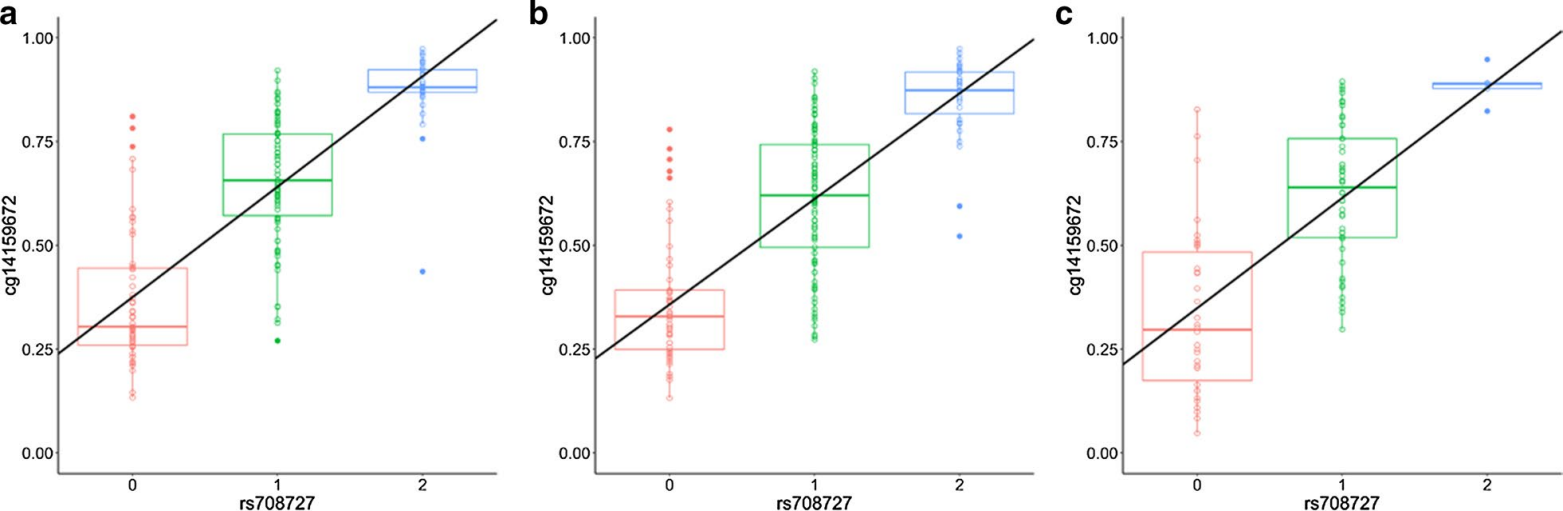
Methylation change as allelic dose of rs708727 changes modeled by *β* values at baseline regressed with allelic dose of rs708727 at one of the representative CpG probes (cg14159672). Scatter plot is colored by the allelic doses of rs708727 where red = 0 (GG), green = 1 (GA), and blue = 2 (AA). An overall linear fit line is also shown. Panel **a** depicts control group, **b** depicts MCI group, and **c** depicts AD group

The effects on the methylation values ($\beta = \frac{M}{M + U + \alpha }$, where *M* and *U* are the methylated and unmethylated signal intensities, and α is an offset) in the DMR regions from alternate allele doses of the two SNP sites were quantitatively evaluated by linear regression at the baseline. We stratified the cohort by diagnostic status during the full sampling time frame by AD only (87 subjects), MCI only (174 subjects), control only (162 subjects) and converters (117 subjects), and effect was evaluated for the three stable diagnosis groups, respectively. We observed that in all diagnostic groups, rs708727 had the most prominent effect as expected (*p* < 2e−16 in control and MCI, *p* < 0.0001 in AD), while rs960603 was not significant for any of the 3 groups (*p* > 0.1) (Table 3, Fig. 3, Additional file 2: Table S2). The slopes of rs708727 were comparable in all diagnostic groups (*p* > 0.05 by testing the differences of the slopes). For both control and MCI groups, age and sex were not significantly associated (*p* > 0.05), while in AD, sex plays a role in the methylation change (*p* < 0.05 in 6 probes, *p* < 0.1 in 9 probes in total), where female shows higher *β* values.

**Table 3:** Model summary for the allelic dosage effects of rs708727 on the *β* values at one of the representative CpG probes (cg14159672)

*dx*	Effects						Model statistics	
	variant	Estimate	SE	*t* value	Pr( >|*t*|)	Signif. codes		
Control	(Intercept)	0.1745	0.1522	1.15	0.254		Residual SD	0.1444
	rs708727	0.2662	0.0218	12.23	< 2E−16	***	Multiple *r*^2^	0.6267
	rs960603	−0.0120	0.0229	−0.52	0.602		Adjusted *r*^2^	0.6172
	Age	0.0031	0.0019	1.66	0.100		*F*-statistic	65.9 (4/157 DF)
	Sex	−0.0165	0.0230	−0.72	0.476		*p *value	< 2.2E−16
MCI	(Intercept)	0.3613	0.1437	2.51	0.013	*	Residual SD	0.1532
	rs708727	0.2544	0.0228	11.17	< 2E−16	***	Multiple* r*^2^	0.5468
	rs960603	−0.0125	0.0226	−0.55	0.580		Adjusted *r*^2^	0.536
	Age	0.0001	0.0019	0.03	0.973		*F*-statistic	50.97 (4/169 DF)
	Sex	0.0030	0.0239	0.13	0.901		*p* value	< 2.2E−16
AD	(Intercept)	−0.0555	0.2317	−0.24	0.811		Residual SD	0.173
	rs708727	0.2654	0.0455	5.84	1.02E−07	***	Multiple *r*^2^	0.5096
	rs960603	0.0016	0.0394	0.04	0.968		Adjusted *r*^2^	0.4857
	Age	0.0036	0.0029	1.27	0.208		F-statistic	21.31 (4/82 DF)
	Sex	0.0874	0.0400	2.19	0.032	*	*p* value	4.49E−12
All stable	(Intercept)	0.1951	0.0941	2.07	0.039	*	Residual SD	0.155
	rs708727	0.2643	0.0150	17.58	< 2e−16	***	Multiple *r*^2^	0.5638
	rs960603	−0.0090	0.0150	−0.60	0.549		Adjusted *r*^2^	0.5597
	Age	0.0020	0.0012	1.69	0.093		*F*-statistic	135.1 (4/418 DF)
	Sex	0.0143	0.0152	0.94	0.348		*p* value	< 2.2E−16

Signif. codes: 0 ‘***’ 0.001 ‘**’ 0.01 ‘*’ 0.05 ‘.’ 0.1 ‘’ 1

### Longitudinal data analysis

**Fig. 4:**
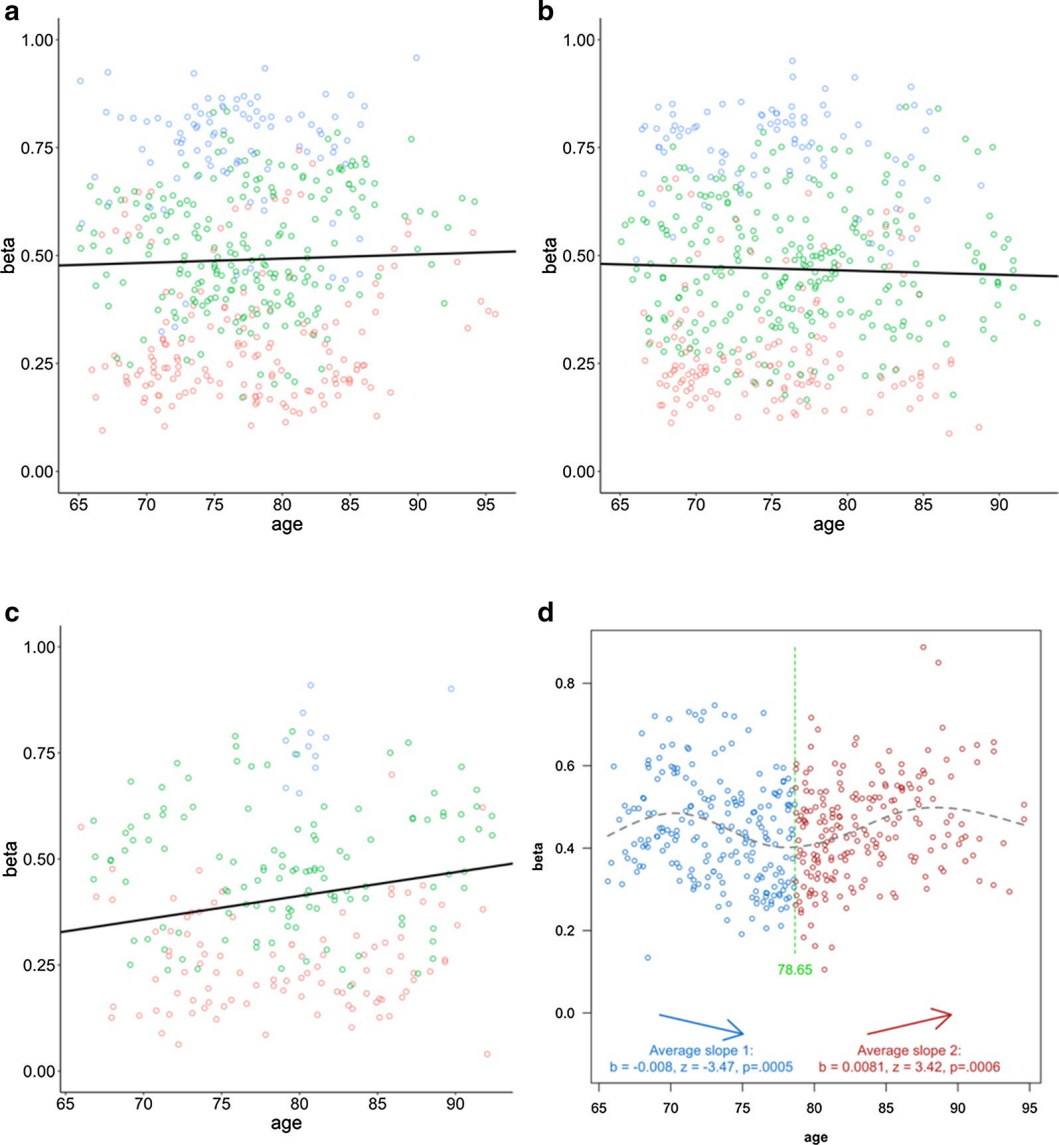
Methylation change as disease/age progresses modeled by *β* values regressed with age at one of the representative CpG probes (cg14893161). Scatter plot is colored by the allelic doses of rs708727 where red = 0 (GG), green = 1 (GA), and blue = 2 (AA) in panels **a**–**c**. An overall linear fit line is also shown. Panel **a** depicts control group, **b** depicts MCI group, and **c** depicts AD group. Panel **d** depicts all the conversion cases where a U-shape is fit and a break point is marked

To evaluate longitudinal changes in methylation levels, we first stratified the cohort by diagnosis status. For stable diagnosis groups (AD/MCI/control), *β* values were fit to age, with sex, allelic dosages for rs708727 and rs960603 as fixed effect covariates by LME models. Consistent with the baseline data, allelic dose for rs708727 demonstrates the most dominant effect on the methylation levels in all the groups (*p* < 2.2e−16), while that of rs960603 is not significant (*p* > 0.1). On top of the effect of allelic dose of rs708727, it also shows an increasing trend of the slope from control to MCI, then to AD (AD vs control, *p* < 0.1 at 4 probes). For both MCI and control groups, no other variables show any effects on any of the probes (*p* > 0.1). In contrast, in AD patients, after controlling allelic dose effect, at least half of the probes (4 probes with *p* < 0.05, 6 probes with *p* < 0.1) still show significant age-dependent methylation elevations (Table 4, Fig. 4a–c, Additional file 3: Table S3). Also consistent with the model of allelic dosage effect at baseline, a majority of the probes (6 probes with *p* < 0.05, 9 probes with *p* < 0.1) also show sex-dependent methylation elevation. Another interesting finding is the weak positive correlation between methylation level and p-tau181 values in plasma (3 probes with *p* < 0.05, Additional file 3: Table S3) as well as the sex effect (3 probes with *p* < 0.05, 6 probes with *p* < 0.1).

**Table 4:** Model summary for the LME modeling between age and the *β* values at one of the representative CpG probes (cg14893161). Fixed effects from age, sex, allelic dosages of the two SNPs and random effects from subject (RID), chip slide and array are shown

*dx*	Fixed effects							Random effects			
	Variant	Estimate	SE	*df*	*t* value	*Pr*( >|*t*|)	Signif. codes	Groups	Name	Variance	SD
Control	(Intercept)	0.2544	0.1185	312.70	2.15	0.0326	*	RID	(Intercept)	0.0154	0.1239
	Age	0.0010	0.0014	340.10	0.68	0.4948		Slide	(Intercept)	0.0030	0.0544
	Sex	−0.0299	0.0218	158.80	−1.37	0.1715		Array	(Intercept)	0.0000	0.0025
	rs708727	0.2392	0.0203	154.90	11.76	< 2E−16	***	Residual		0.0017	0.0408
	rs960603	0.0018	0.0215	159.60	0.08	0.9339					
MCI	(Intercept)	0.2973	0.1064	351.20	2.795	0.00547	**	RID	(Intercept)	0.0123	0.1108
	Age	−0.0010	0.0013	385.40	−0.710	0.4782		Slide	(Intercept)	0.0089	0.0943
	Sex	0.0071	0.0221	168.30	0.321	0.7482		Array	(Intercept)	0.0001	0.0077
	rs708727	0.2676	0.0210	167.00	12.742	< 2E−16	***	Residual		0.0014	0.0374
	rs960603	−0.0038	0.0206	165.30	−0.187	0.8520					
AD	(Intercept)	−0.2992	0.1659	117.40	−1.803	0.07391		RID	(Intercept)	0.0156	0.1250
	Age	0.0056	0.0020	122.80	2.775	0.00638	**	Slide	(Intercept)	0.0081	0.0899
	Sex	0.0696	0.0351	83.48	1.983	0.0506		Array	(Intercept)	0.0000	0.0050
	rs708727	0.2722	0.0379	69.96	7.179	5.90E−10	***	Residual		0.0019	0.0432
	rs960603	0.0006	0.0335	77.22	0.019	0.9849					
All stable	(Intercept)	0.0996	0.0715	798.00	1.391	0.1645		RID	(Intercept)	0.0132	0.1150
	Age	0.0015	0.0009	864.10	1.733	0.0835		Slide	(Intercept)	0.0080	0.0897
	Sex	0.0089	0.0144	442.00	0.622	0.5343		Array	(Intercept)	0.0000	0.0054
	rs708727	0.2831	0.0128	340.10	22.051	< 2e−16	***	Residual		0.0017	0.0408
	rs960603	0.0038	0.0131	374.50	0.288	0.7733					

Signif. codes: 0 ‘***’ 0.001 ‘**’ 0.01 ‘*’ 0.05 ‘.’ 0.1 ‘’ 1

To test if there is a turning point for the methylation level of PM20D1 in the disease progression process, we employed the U-shape test by the two-lines method on the longitudinal data for the converters group (117 subjects). We adopted the ‘two-lines’ U-shape test approach to demonstrate (and statistically test) that there is a non-monotonical trajectory in the methylation level as a function of disease progression, by characterizing the nonlinearity without making functional-form assumptions about *f*(*x*). This approach has also been adopted in the AD research field previously to model disease progression [19]. For each probe, a clear U shape was observed (*p* < 0.01 on both slopes), with the breakpoint identified at ~ 78 to 79 years of old (Additional file 3: Table S3, Fig. 4d). Allelic doses of rs708727 and rs960603 also show significant effect on the correlation, although the former is more prominent than the later. Consistent with our previous analysis, we also detected a sex effect in the majority of the probes (5 probes with *p* < 0.05, 7 probes with *p* < 0.1, *t* test).

### Correlation of methylation data with gene expression

**Fig. 5:**
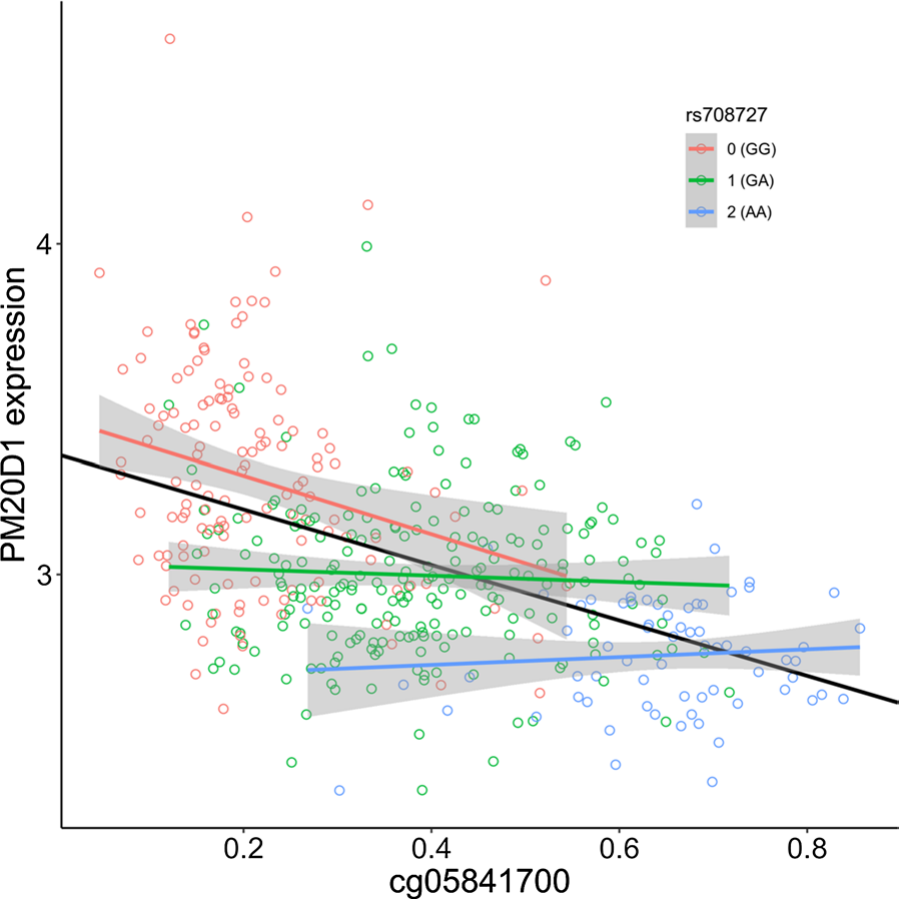
Correlation of methylation *β* values at one of the representative CpG probes (cg05841700) with gene expression of PM20D1 for the ADNI cohort. An overall fit line and the fit lines stratified by the allelic doses of rs708727 are shown

To characterize associations between methylation changes and expression of PMD20D1, we integrated available DNA methylation data from subjects with a stable diagnosis, with matched whole blood gene expression data. For all 10 probes, we observed an inverse correlation between probe methylation *β* value and PM20D1 expression (Additional file 4: Table S4, Fig. 5), with *p* < 0.001 at 6 probes and *p* < 0.01 at 8 probes. We again observed a significant association between dosage of rs708727 and PM20D1 expression (*p* < 0.0001, *t* test), while that of rs960603 shows negligible effect (*p* > 0.1, t test). We did not observe associations between sex, age or diagnosis and PM20D1 gene expression.

Strikingly, we observed that the observed association between cg05841700 and PM20D1 was entirely driven by subjects with an allelic dose of 0, homozygous GG at rs708727. When the correlation is stratified by allelic doses of rs708727, it is apparent that only in the hypomethylated samples (allelic dose = 0, homozygous GG) there is a significant linear correlation (Fig. 5, red line and dots). There is no such linear correlation in the heterozygous samples (allelic dose = 1, heterozygous GA, Fig. 5, green line and dots), or even less so in the hypermethylated samples (allelic dose = 2, homozygous AA, Fig. 5, blue line and dots). This is consistent with the recent discovery that the methylation level of PM20D1 promoter cannot be directly translated into gene expression, due to blockage of an enhancer downstream for the hypermethylated groups (AD-risk associated haplotype). Only from individuals with unmethylated PM20D1 where the enhancer region physically interacts with the promoter, can PM20D1 transcription begin [13]. The current study provides numeric representations of the relationship and confirms a similar observation that is made in a peripheral tissue (blood) in comparison with the findings originally reported in brain tissue samples [13].

### Data from brain tissue support findings in whole blood

**Fig. 6:**
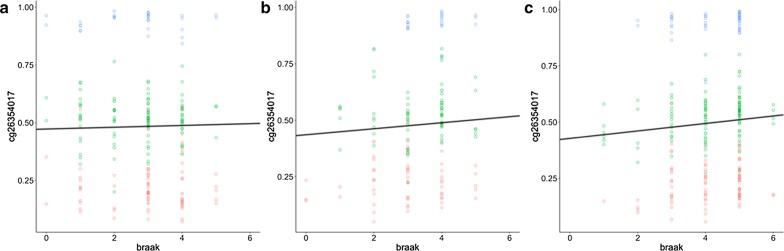
Methylation change as disease progresses modeled by *β* values regressed with Braak score at one of the representative CpG probes (cg26354017) from the ROSMAP brain samples. Scatter plot is colored by the allelic doses of rs708727 where red = 0 (GG), green = 1 (GA), and blue = 2 (AA). An overall linear fit line is also shown. Panel **a** depicts control group, **b** depicts MCI group, and **c** depicts AD group

As a validation of the methylation alteration at the PM20D1 promoter region observed in blood-based data, we investigated DNA methylation changes at 6 CpG sites profiled from postmortem brain prefrontal cortex tissue samples collected as part of the ROSMAP cohort. We observed a strong association between CpG methylation and Braak staging (*p* < 0.05) in participants with an AD diagnosis (Table 5, Additional file 5: Table S5, Fig. 6). We did not observe any association in the control group for any of the 8 CpG sites, and only one probe was found to be significantly associated with Braak staging in the MCI group (*p* = 0.038 at cg24503407), and another marginally significant (*p* = 0.070 at cg17178900). When combined together, there is a linear correlation (*p* < 0.05) between methylation level and Braak score at 6 out of the 8 probes for all the subjects. This correlation could be attributed to the larger sample size, AD subgroup and wider range of Braak stages in the overall group. Again, rs708727 was found to be a highly significant (*p* < 2e−16) covariant of all probes, while rs960603 is not. In contrast with our previous analysis, we did not observe an association with sex. Interestingly, the correlation between DNA methylation levels and pathological biomarkers (amyloid and tangles) shows a positive association of quantitative overall amyloid level at 3 probes in the AD subgroup, 1 probe in the MCI subgroup and 0 in the controls out of the 8 probes in total. For tangles, there is no association of PM20D1 methylation at all for any of the diagnostic subgroups, but a weak association in the overall group (*p* < 0.05 for 4 out of 8 probes, Additional file 5: Table S5). We also observed an inverse linear correlation between *β* value and PM20D1 expression (Additional file 6: Table S6, Additional file 9: Fig. S1). Consistent with our findings within the blood-based ADNI data, after stratification by rs708727 genotype, the observed linear correlation was primarily attributed to the lower AD-risk populations (rs708727 allelic dose = 0, GG).

**Table 5:** Model summary for the effects of Braak staging on the *β* values at one of the representative CpG probes (cg26354017)

*dx*	Effects						Model statistics	
	variant	Estimate	SE	*t* value	*Pr*( >|*t*|)	Signif. codes		
Control	(Intercept)	0.3302	0.1194	2.767	0.006	**	Residual SD	0.1008
	Braak	0.0039	0.0064	0.602	0.548		Multiple *r*^2^	0.8457
	rs708727	0.3340	0.0143	23.379	< 2e−16	***	Adjusted *r*^2^	0.8419
	rs960603	0.0095	0.0140	0.682	0.496		F-statistic	226.9 (5/207 DF)
	msex	0.0043	0.0147	0.290	0.772		*p* value	< 2.2E−16
	age_death	−0.0016	0.0014	−1.123	0.263			
MCI	(Intercept)	0.2988	0.1729	1.73	0.086		Residual SD	0.1016
	Braak	0.0133	0.0082	1.63	0.106		Multiple *r*^2^	0.8476
	rs708727	0.3446	0.0182	18.98	< 2e−16	***	Adjusted * r*^2^	0.8423
	rs960603	−0.0203	0.0183	−1.11	0.269		*F*-statistic	159.1 (5/143 DF)
	msex	0.0245	0.0175	1.40	0.164		*p* value	< 2.2E−16
	age_death	−0.0014	0.0020	−0.70	0.483			
AD	(Intercept)	0.2564	0.1636	1.57	0.118		Residual SD	0.092
	Braak	0.0166	0.0056	2.95	0.004	**	Multiple *r*^2^	0.8813
	rs708727	0.3362	0.0146	23.06	< 2e−16	***	Adjusted *r*^2^	0.8788
	rs960603	0.0102	0.0142	0.72	0.472		*F*-statistic	351.9 (5/237 DF)
	msex	−0.0140	0.0130	−1.08	0.282		*p* value	< 2.2E−16
	age_death	−0.0013	0.0018	−0.68	0.496			
All	(Intercept)	0.3262	0.0795	4.10	4.66e−05	***	Residual SD	0.097
	Braak	0.0108	0.0034	3.16	0.0016	**	Multiple *r*^2^	0.8590
	rs708727	0.3376	0.0087	38.79	< 2e−16	***	Adjusted* r*^2^	0.8578
	rs960603	0.0038	0.0086	0.44	0.657		*F*-statistic	729.9 (5/599 DF)
	msex	0.0015	0.0085	0.18	0.861		*p* value	< 2.2E−16
	age_death	−0.0018	0.0009	−1.86	0.063			

Signif. codes: 0 ‘***’ 0.001 ‘**’ 0.01 ‘*’ 0.05 ‘.’ 0.1 ‘’ 1

**Table 6:** Demographic profile for the subjects in the longitudinal data analysis by different diagnosis groups

Demographics	Stable diagnosis			Converters
	Control	MCI	AD	
Total subjects	162	174	87	117
Male/female	79/83	104/70	57/30	68/49
# of visits	2.66 ± 0.69	2.63 ± 0.68	2.10 ± 0.99	3.10 ± 0.88
Age at visits	77.34 ± 6.19	75.78 ± 6.20	79.01 ± 6.62	78.18 ± 6.33
Duration of follow-ups	1.71 ± 0.71	1.73 ± 0.67	1.12 ± 1.00	2.07 ± 0.95

### Direction comparison of DNA methylation between brain tissues and whole blood

The consistent findings between the different brain regions and the peripheral blood from different cohorts prompted a direct comparison between DNA methylation in blood with brain regions for the CpG probes at PM20D1 promoter region, using published results in the third independent cohort (the London cohort). Across the 8 CpG probes from the Methylation 450 K array at PM20D1 promoter region, the positive correlation of methylation between blood and any of the four brain regions is all highly significant, with correlation coefficient ranging from 0.857 to 0.976 (*p* < 0.0001, Additional file 7: Table S7, Additional file 9: Fig. S2). Of note, this correlation is regardless of age, sex or pathological state of the individuals, highlighting a consistent interindividual covariance in whole blood and that observed in all four brain regions, specific to the promoter region of PM20D1. The clear trimodal distribution of DNA methylation suggests that the mQTL mediates much of the observed cross-tissue similarities, and the profile at PM20D1 in blood could be used as a proxy to predict DNA methylation levels in the brain [20].

In summary, our results collectively demonstrate that there is a U-shaped dynamic trajectory in the methylation profile for PM20D1 promoter in the whole blood samples from ADNI cohort, from normal state to MCI then to LOAD. The change trend of the methylation profile at the same promoter in the brain samples from the ROSMAP cohort, and the remarkable correlation between blood and brain and between several brain tissues in the third cohort confirm that this is a universal observation conserved in different tissue types across different demographics.

## Discussions

Early diagnosis and treatment of AD have been called out as the most urgent problem to tackle for this second century disease [21]. Nevertheless, to date not a single easy-to-measure biomarker has been fully established for its diagnosis and it remains elusive to identify blood-based biomarkers that will pinpoint AD in its earliest phases. As the most important goal is to detect AD at the earliest possible stage (pre-dementia) and identify ways to track the disease’s progression with biomarkers, ADNI data provide unique opportunity to observe the drastic change of molecular profiles during disease progression. Data obtained from microarray whole-genome DNA methylation profiling enable large scope in both time frame and sample size offering unprecedented insights into the dynamics of epigenetic signatures during the early phases in a large AD cohort. On the other hand, epigenetic signatures are largely tissue-specific; so understanding how observations obtained from peripheral tissue such as blood might relate to brain tissues needs further examination.

As a pioneering study in exploring the whole dataset, we made a direct comparison of baseline measurements among control, MCI, and AD groups from the whole-genome methylation profiling data. We found that the promoter of PM20D1 gene locus consistently displayed hypomethylation from control to MCI, and even further to symptomatic AD. PM20D1 has recently been reported as an mQTL in two major AD affected brain regions, the hippocampus and the frontal cortex, based on the comparisons between samples from healthy controls and patients with advanced-stage AD, although it is been found to be hypermethylated in the latter [13]. In the report, an allele-dependent correlation with PM20D1 promoter methylation is identified for the rs708727–rs960603 haplotype. In the proposed mechanistic model, PM20D1 has been suggested as a protective gene of AD, whose elevated expression levels might provide a potential cellular defense mechanism. For AD non-risk-haplotype carriers, specifically those with homozygous reference allele haplotype at both SNP rs708727 (GG) and rs960603 (CC), PM20D1 methylation level is lower; in the presence of AD-related stress, expression is enhanced to reduce ROS-induced cell death and Aβ levels and prevent memory impairment. In contrast, in samples with hypermethylated PM20D1 (high risk, homozygous alternate allele haplotype carriers, AA/TT), the promoter region is not contacted by the enhancer and transcription does not occur, which results in low PM20D1 expression, and there is no protective effect against Aβ. The role of PM20D1 in AD has since then been further explored [22, 23], showing that it is the sole risk gene with consistently enriched promoter hypermethylation in AD patients, and upregulated by Aβ and reactive oxygen species, and being neuroprotective when overexpressed in cell and primary cultures.

In concordance with the previous mechanistic model, we first validated the allelic dosage effects on PM20D1 promoter region in the blood samples, but found it is only due to the genotypes of rs708727. The allelic dosage of rs708727 has been found to be highly significant in determining the methylation level of the CpG sites, with higher dosage associated with higher baseline methylation quantitatively. Notably, this QTL has an effect size of ~ 25% (Fig. 3, Table 3). Although not most common, such effect size of a QTL has been reported in multiple studies, e.g., [24, 25]. In fact, this agrees almost perfectly with what is been observed in brain tissues as reported in the aforementioned work of Sanchez-Mut et al. [13, 22]. Interestingly, although rs960603 is reported to be co-segregated as a haplotype in nearly 85% of cases in the 1000 Genomes Project, its allelic dosage is not as significant in multiple tests within our analysis. This is in agreement with a most recent linkage disequilibrium analysis showing in the haplotype, six SNPs (rs17772159, rs823074, rs803275, rs9438393, rs823090 and rs17772143) but not rs960603 were tightly linked to the lead QTL SNP rs708727 [26]. Our study thus confirms that PM20D1 is an mQTL mediated primarily by the AD-risk associated SNP (rs708727) as measured in peripheral blood. Furthermore, we exploited the longitudinal data and demonstrated that hypomethylation actually occurs before the symptomatic onset of the disease, conceivably to facilitate increasing expression of the gene to activate its protective function. As disease progresses, methylation level is gradually elevated in most of the CpG sites in the AD patients, which ultimately leads to depletion of the gene transcription and expression. This phenomenon is not observed in the control or MCI groups as their methylation levels are almost constant (Fig. 4). This explains the previously observed hypermethylation in late stage AD patients (Braak staging ≥ 5) in comparison with control. Our models provide a comprehensive picture of the dynamic change of the methylation profile at PM20D1 promoter region, thus complementing the previous work [13]. Of note, the initial methylation decline for these probes actually prevails in AD vs control for all the stratified genotypes of rs708727 (Additional file 8: Table S8), indicating constant hypomethylation of the promoter region at the early stages of the disease regardless of the patient’s disease risk, although it seems that for the AD patients carrying the high-risk SNP, the methylation elevation is faster compared with those with lower risk (Additional file 9: Fig. S3). In our LME model, random slopes of age are not tested due to limited data points per individual which would result in singular fit, or failed converge of the random slope model. By excluding the random slope for the priming manipulation, we assume that the priming effect of age (disease progression) is invariant across subjects in the population. This might be oversimplified and there is possibility that different risk groups possess different rates of increasing methylation level across the PM20D1 promoter region. This hypothesis cannot be directly tested in the current dataset due to the small sample size of AD patients carrying the high-risk SNP (*n* = 5), but it warrants further exploration with larger datasets in the future.

From the U-shape test for the subjects with converted diagnosis, it is identified that 78–79 years old would be the turning point for the methylation level of the probes. This matches the average initial diagnosis age for LOAD at ~ 78 years old in the general population [27]. Whether the turning point is triggered by some other factors or solely determined by age is something that requires further investigation. Furthermore, we found a sex-dependent effect on the methylation elevation for most of the probes, indicating that female is at higher risk for hypermethylation of PM20D1 promoter. This reveals yet another possible contributing factor to the females’ higher odds for AD [28, 29].

Despite this, hypomethylation does not necessarily translate into higher gene expression. The correlation between methylation profile and gene expression of PM20D1 from cross-sectional data clearly demonstrated that only within the lower-risk genotype carriers, there is an inverse linear relationship. Higher risk genotype carriers’ expression profiles are not correlated with methylation, which can be attributed to the inaccessibility of the enhancer to the gene promoter, as suggested by the mechanism model for PM20D1 function in AD. These findings indicate that methylation signatures at the PM20D1 locus are more robustly associated with conversion to AD, than PM20D1 expression.

The methylation elevation trend after the onset of AD has been once more observed in brain tissues in the ROSMAP cohort, by using Braak stage as a proxy of the disease’s progression. The same correlation pattern between methylation and gene expression for PM20D1 in the brain tissues, as well as the remarkable direct correlation between blood and brain tissue’s methylation levels in the third independent cohort at the CpG sites provide strong evidence of a link between peripheral blood methylation profiles and AD-associated methylation differences in the brain tissues at PM20D1 region. Although sex’s effect has not been repeated in the brain tissues probably due to the fact the ROSMAP data has been adjusted for age and sex [30], our work illustrates that blood methylation at the PM20D1 promoter region could potentially serve as a surrogate for brain methylation for the study of epigenetics of this gene for AD pathology, along the course of disease evolution.

Of special note, we have also attempted the correlation between PM20D1 methylation level and a few pathological biomarkers, in order to better understand the significance of PMD20D1 methylation in the evolution of AD pathology. For the ROSMAP cohort, methylation level of PM20D1 might be positively associated with amyloid in AD patients (*p* < 0.05 for 3 out of 8 probes in total), but not in MCI (1 probe), nor control (0 probe) or the overall group (1 probe) (Additional file 5: Table S5). For tangles, there is no association of PM20D1 methylation in any of the three diagnostic subgroups, but a weak association in the overall group (*p* < 0.05 for 3 out of 8 probes), probably due to larger sample size and wider range of tangle scores. In the ADNI cohort, there is no correlation with any of the biomarkers (Aβ1-42, t-tau and p-tau181) from cerebral spinal fluid (CSF) in any of the three diagnostic subgroups, or the overall group (results not shown, biomarker data from [31]), but some positive correlations were found for p-tau181 in plasma, for AD patients only (*p* < 0.05 at 3 probes, Additional file 3: Table S3). This is in line with the report that plasma p-tau181 has greater variability than CSF p-tau181 and may be differently regulated depending on Aβ status [32]. Recently, it has been demonstrated that plasma ptau-181 is an indicator of very early brain amyloidosis [33], so the findings imply that PM20D1 could play a role in the metabolism of amyloid proteins, showing correlation with Aβ biomarkers, but not quantitative tangle scores. Another possible scenario is that soluble amyloid peptide, or other secondary factors associated with very early amyloidosis in the brain, actually triggers hypomethylation of PM20D1 in AD patients, and insoluble plagues in AD stages further affects its methylation level, so the correlation is observed with Aβ deposition.

As a circulating enzyme, PM20D1 regulates a class of N-lipidated amino acids in vivo, and these metabolites function as endogenous uncouplers of mitochondrial respiration [34]. It has been implicated in obesity, type 2 diabetes [34], pain [35] and more recently, Alzheimer's disease [13]. In mouse models, PM20D1 activity was dramatically increased in lipoprotein fractions from APOE-knockout (KO) mice versus wild-type mice. The activation of the PM20D1/N-acyl amino acid pathway is suggested as a contributor to the protection from metabolic and neurological diseases observed in APOE-KO mice [36]. The recent study focusing on PM20D1′s role in AD demonstrated that PM20D1 expression is increased both in vitro and in vivo following neurotoxic insults, probably by activating the hypomethylation machinery as illustrated in our work. Forced overexpression of PM20D1 in the hippocampus results in improved learning performance in the mouse model of AD, whereas PM20D1 knockdown increases amyloid plaque load, so it has been suggested to have a protective role against AD. Given that LOAD has also been suggested as a metabolic disorder [37–39], the interplay of PM20D1, more specifically how it operates a protective function against AD, together with other genes implicated in the metabolism for LOAD could help advance our understanding of the disease, and subsequently more efficient hunting for therapeutics.

Accurate, minimally invasive and timely diagnosis of probable AD in living individuals has always been challenging, although most recently great breakthroughs are being made in blood-based biomarkers, such as p-tau181 [32] and p-tau217 [40, 41]. Multiple factors contribute to it, such as heterogenicity of the disease, inaccessibility of the pathological tissues, and lack of robust and readily measurable biomarkers [6]. The dynamic methylation alteration for PM20D1, depicted by the longitudinal data with individuals both before disease onset and following clinical diagnosis opens a probable channel to monitor the disease, and the possibility of a diagnostic tool. Notably, the initial methylation decrease is universal to all the risk-associated SNP genotypes (Additional file 8: Table S8), highlighting its application potential regardless of the genotypes of the SNP locus, thus associated disease risk. The random effect from LME modeling shows that intra-individual methylomic variation at PM20D1 (SD > 0.1 for RID) is still non-negligible after controlling fixed effects from age, sex, allelic dosage of rs708727 and rs960603. Notably, APOE status has been initially considered as a covariant in all of our studies, but the effect is not significant (*p* > 0.1), thus not included in the final analysis. While there are presumptively still other factors underlining the baseline methylomic *β* values for PM20D1 promoter, the overall trend of individual methylation change, instead of absolute methylation level (which is primarily determined by the SNP allelic dose), is worthy of further evaluation. The current study is the first of its kind applying large-scale longitudinal data to obtain novel insights into the epigenetic signature of PM20D1 intervening with LOAD. Further in-depth study, including those in animal models and patients, as well as its precise relationship with Aβ proteins and p-tau levels in blood could help define its sensitivity and specificity to differentiate AD from non-AD and its broader utility down the road.

The preliminary result we observed in the longitudinal data from ADNI cohort is just one piece of evidence that there is correlation for specific pathological changes in AD between different tissues from different cohorts, as manifested at PM20D1 locus. An expansive analysis that addresses DMR and longitudinal data in an integrated model would reveal even more exciting findings; nevertheless, it requires tremendous computational resources and innovative methodologies and tools, which is worth active pursuing. The current study exemplified utilization of the data to distill useful information in the epigenetic landscape of AD. Further exploitation is thus warranted and would be beneficial to the early diagnosis and effective treatment of the formidable disease.

## Conclusions

The longitudinal data from ADNI methylation profiling present compelling evidences demonstrating that changes to the methylome in LOAD detected in blood could reflect pathologic processes implicated in ongoing neurodegeneration in the brains. Specifically to PM20D1 gene locus, it clearly confirms PM20D1 is a methylation QTL essentially coupled to an AD-risk associated SNP rs708727, and illustrates its dynamic role of being hypomethylated in the conversion phase and gradually turning into hypermethylation after onset and during progression of the disease. Our results call for further study, both for PM20D1′s role in AD pathology and its potential as a blood-based biomarker, to address the urgent need of early detection and treatment of AD.

## Methods

### Study cohort and data source

ADNI data were all downloaded from the ADNI database (http://adni.loni.usc.edu/). The ADNI was launched in 2003 as a public–private partnership, led by Principal Investigator Michael W. Weiner, MD. The primary goal of ADNI has been to test whether serial magnetic resonance imaging (MRI), positron emission tomography (PET), other biological markers, and clinical and neuropsychological assessment can be combined to measure the progression of mild cognitive impairment (MCI) and early Alzheimer's disease (AD). For up-to-date information, see www.adni-info.org. For details of the methylation profiling procedure, refer to the documentation on the genetic data download page. Briefly, DNA methylation profiling was performed at AbbVie from a total of 1,920 blood samples, including 1719 unique samples and 201 technical replicates (653 unique individuals). Longitudinal DNA samples at baseline and follow-ups were obtained from the cohort. The Illumina Infinium HumanMethylationEPIC BeadChip Array (www.illumina.com), which covers ~ 866,000 CpGs, was used for methylation profiling. After multiple steps of quality check (QC), 1905 samples from 649 subjects were retained and raw methylation data (idat files) were released and analyzed in our study.

**Fig. 7:**
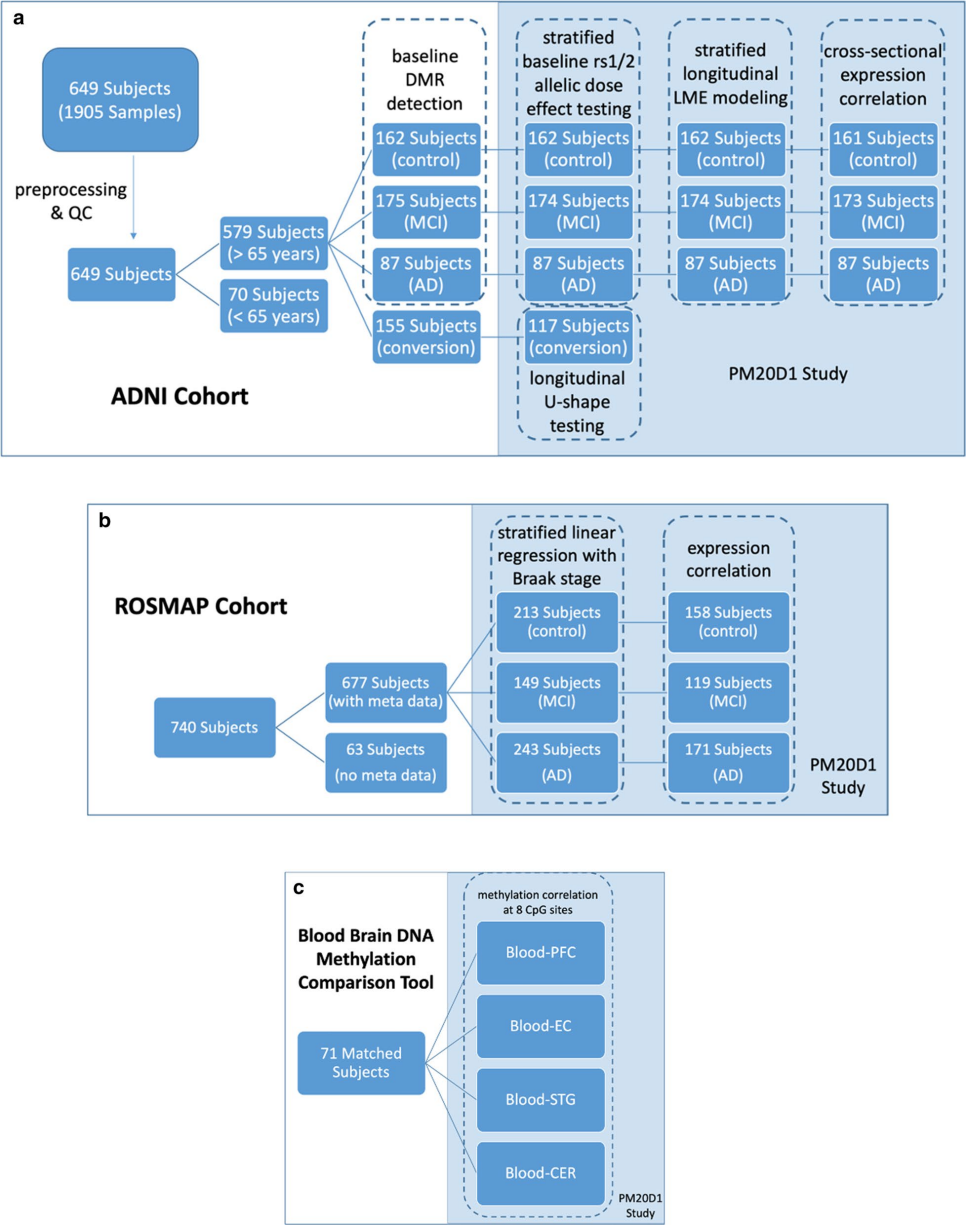
Workflow and subject selection for the study outlined in this work

Genotype data were obtained from ADNI whole-genome sequencing (WGS) panel, where genotypes were called by the ADNI genetics core using the pipeline following Broad best practices (BWA [42] and GATK HaplotypeCaller [43, 44]). Gene expression data were obtained from blood samples from the 811 participants in the ADNI WGS cohort. The Affymetrix Human Genome U219 Array (Affymetrix (www.affymetrix.com), Santa Clara, CA) was used for expression profiling. The processed gene expression profile was downloaded and used for analysis. Diagnosis data and other demographics (sex, date of birth) were also obtained from ADNI database. Subject selection for the different study sections and the workflow of the ADNI cohort are outlined in Fig. 7a).

All the data from ROSMAP cohort [45, 46] were downloaded from Accelerating Medicines Partnership-Alzheimer’s Disease (AMP-AD) Knowledge Portal following required guidelines. Methylation *β* values at the interesting CpG probes were obtained from the methylation profile generated on prefrontal cortex samples collected from 740 individuals using the Illumina HumanMethylation450 BeadChip [7]. Genotypes at the interesting loci were extracted from the published WGS data from 1196 subjects [30]. Gene expression Fragments Per Kilobase of transcript per Million mapped reads (FPKM) values were obtained from bulk RNAseq data, where samples were taken from the gray matter of the dorsolateral prefrontal cortex of 724 subjects [30] and the data for 640 subjects were available for download. Clinical and neuropathology data including final diagnosis and Braak stages were matched for each individual and used in the analysis. Subject selection for the different study sections and the workflow of the ROSMAP cohort are outlined in Fig. 7b).

### ADNI methylation data quality control and normalization

Raw DNA methylation data (idat files) were imported into R, and the Bioconductor package ‘bigmelon’ [47] was used for initial data processing. Detection p values (detP), methylation (M) and unmethylation (uM) intensities and *β* values defined as the ratio between the methylated and total signals (M + uM) were calculated and exported. The ChAMP pipeline [48] for EPIC array was then used for all the downstream analysis following the default workflow. Multiple filtering steps were first applied, by excluding probes with detection *p* value (default > 0.01), probes with < 3 beads in at least 5% of samples per probe, all non-CpG probes contained in the dataset [49], all SNP-related probes, all multi-hit probes and all probes located in sex chromosomes [50]. Data were then normalized with BMIQ method [51], and batch effects from chip slide were corrected by ComBat [52]. Cell proportion was calculated based on a reference DNA methylation profile, and cell type influence on the whole blood data was removed by RefbaseEWAS [53]. The ‘estimateCellCounts’ function in minfi [54] was used to estimate the proportional abundance of blood cell types in the each sample based on the intensity of specific for inter-group comparisons as probes present in the EPIC array.

### Differential methylated region (DMR) detection

Since the longitudinal data consist of 649 subjects sampled at dates spanning up to 5 years with 390 subjects having at least 3 visits’ data, many of the subjects have changed their diagnostic status over the time course. To focus on the study of LOAD, 70 subjects with sampling age < 65 at any time point were removed. The diagnosis dates were compared against the blood sample collecting dates for each of the remaining 579 subjects, and the closest diagnosis in time (< 0.5 years) was chosen as the diagnosis for each individual sample. For clear DMR detection, only those samples keeping a stable diagnosis (i.e., control, MCI, or AD across all the time points for each subject, respectively) were retained for analysis. This left 424 total subjects for the DMR detection. Among them, 162 were control, 175 were MCI, and the remaining 87 were AD patients. DMRs were detected using the baseline *β* values by the BumperHunter method implemented in ChAMP [55]. When technical replicates exist for the same sample, one data point was randomly picked. Comparisons were made for AD vs control, AD vs MCI and MCI vs control, respectively.

### Allelic dosage effect in the DMR region at baseline

Out of the 424 subjects with stable diagnosis, 423 were genotyped by WGS. Their baseline measurements used for DMR detection were also used for modeling the dosage effect of the two reported associated SNP rs708727 (GG = 0, GA = 1, and AA = 2) and rs960603 (CC = 0, CT = 1 and TT = 2). Their effects to the *β* values of the probes at the promoter regions of the PM20D1 gene locus were modeled by the lm function in R, using age, sex as covariates, stratified by the diagnosis group. The formula, in R syntax, for the model is:

$$\beta \sim rs708727 + rs960603 + age + sex$$

### Longitudinal data modeling

Longitudinal analysis was performed on the whole longitudinal data for 540 total subjects with genotypes available from the WGS panel (423 stable diagnosis, 117 converted cases). Their demographics profile is reported in Table 6. For the subjects with a stable diagnosis, linear mixed-effects (LME) models were fit for the *β* values for the probes at the promoter regions of the PM20D1 gene locus, for the 162 controls, 174 MCI patients and 87 AD patients, respectively, by the lmer function in the R package ‘lmerTest’ [56]. The formula for the linear mixed-effects model is:

$$\beta \sim age + sex + rs708727 + rs960603 + \left( {1{|}RID} \right) + \left( {1{|}Slide} \right) + (1|Array)$$

where age, sex and the allelic dosages for the two known SNP rs708727 and rs960603 were modeled as fixed effects, and subject ID (RID), array and slide were modeled as random effects.

The exam dates from plasma p-tau181 data (obtained from recent ADNI release http://adni.loni.usc.edu/new-longitudinal-plasma-p-tau181-results-available/) were matched against the dates for sample collection of the methylation array, and data from 412 subjects (159 controls, 174 MCI and 79 AD patients) were fit by LME model using the formula:

$$\beta \sim ptau + sex + rs708727 + rs960603 + \left( {1{|}RID} \right) + \left( {1{|}Slide} \right) + (1|Array)$$

where ptau is log transformed and modeled as fixed effect.

For the group of 117 subjects with changed diagnosis, a U-shape test was carried out by the two-line method introduced by Simonsohn [57] and using the code (http://webstimate.org/twolines/twolines.R), testing if age has a U-shaped effect on *β* values, controlling for sex, allelic dosages of rs708727 and rs960603. The breakpoint is set using the "Robin Hood" algorithm, seeking to obtain higher power to detect a U-shape if it is present [57].

### Methylation correlation with gene expression

For the 423 subjects with stable diagnosis (as well as genotype data), gene expression data were also available from ADNI microarray profiling. All the gene expression data were cross-sectional, and only information on sample collection years is available. The sample collection years were matched against the methylation profiling sample collection years, and those samples collected in the same year were deemed as a matching expression-methylation data pair for each subject. The collection years were more than one year apart between methylation and expression samples for two subjects; thus, they were excluded from this analysis. The relationship between gene expression of PM20D1 and the *β* value for each probe was modeled for the 421 data pairs by the lm function in R, using age, sex, diagnosis and allelic dosages of rs708727 and rs960603 as covariates, following the equation:

$$e \sim \beta + age + sex + dx + rs708727 + rs960603$$

### Methylation data analysis for ROSMAP cohort

Subjects’ clinical diagnosis, Braak stages for brain tissues and demographic information including sex and age at death were obtained from AMP-AD portal. A total of 677 subjects were found to have genotypes, Braak stages and methylation profiles available. Only those subjects with diagnosis as no cognitive impairment (control) or MCI/AD without any other cause of cognitive impairment (cogdx = 1, 2 or 4), totaling 605 subjects were included in the final analysis. They were stratified by the diagnosis, and linear regression models in respect to Braak stage, (log transformed) amyloid and (log transformed) tangles were built for the *β* values of the 8 CpG probes at the promoter regions for PM20D1, respectively, controlling for age, sex, allelic dosages of rs708727 and rs960603, using the lm function in R. Gene expression data were also matched with the methylation data, and 448 subjects out of the 605 were found to have gene expression data from RNASeq profiling. Linear regression models were built between gene expression FPKM value of PM20D1 and the *β* value at each probe for the 448 data pairs by the lm function in R, using age, sex, diagnosis and allelic dosages of rs708727 and rs960603 as covariates.

### DNA methylation correlation between brain tissue and whole blood

We utilized the Blood Brain DNA Methylation Comparison Tool (https://epigenetics.essex.ac.uk/bloodbrain/), which allows systematic investigation of the correlation of DNA methylation in blood with four brain regions (prefrontal cortex, entorhinal cortex, superior temporal gyrus and cerebellum) from 71 to 75 matched samples in the MRC London Neurodegenerative Disease Brain Bank [8] for all probes present on the Illumina 450 K Beadchip array [17]. The cohort included both neuropathologically unaffected controls and individuals with variable levels of neuropathology. The data are from published results in 4 dissected brain regions (PFC: *n* = 114, EC: *n* = 108, STG: *n* = 117 and CER: *n* = 112) and matched premortem whole blood samples (*n* = 80) from an overlapping set of 122 individuals [17]. All the correlation values were obtained directly from the database.

## Supplementary information

**Additional file 1**. **Table S1**: The differentially methylated regions (DMRs) as detected by the comparisons among control, MCI and AD. Each tab shows the significant regions as ranked by the p values for each comparison.

**Additional file 2**. **Table S2**: P values and significant slopes for the regression lines modeled between alternate allelic doses of rs708727/rs960603 and β values for the 10 CpG probes at PM20D1 promoter region in the different diagnosis groups.

**Additional file 3**. **Table S3**: P values and significant slopes for the regression lines modeled by linear mixed effect modeling between age or p-tau181 and β values for the 10 CpG probes at PM20D1 promoter region in the different diagnosis groups.

**Additional file 4**. **Table S4**: P values for the regression lines between the β values of the 10 CpG probes and the gene expression of PM20D1.

**Additional file 5**. **Table S5**: P values for the regression lines between Braak score, amyloid or tangles and β values for the 8 CpG probes at PM20D1 promoter region in the different diagnosis groups from the ROSMAP cohort brain samples.

**Additional file 6**. **Table S6**: P values for the regression lines between the β values of the 8 CpG probes and the gene expression of PM20D1 from the ROSMAP cohort brain samples.

**Additional file 7**. **Table S7**: P values and regression coefficients for the correlation between DNA methylation in blood with four brain regions (prefrontal cortex, entorhinal cortex, superior temporal gyrus and cerebellum) from 71 to 75 matched samples for all the 8 CpG probes. The data are taken from the Blood Brain DNA Methylation Comparison Tool [17].

**Additional file 8**. **Table S8**: Mean β values and their standard deviations (SD) at baseline measurements for the sex and age matched samples at the 10 CpG probes stratified by the allelic dose of rs708727. Control and MCI samples were obtained by matching AD samples’ sex and age using ‘matchControls’ command in the R package ‘e1071.’

**Additional file 9**. **Figure S1**. Correlation of methylation β values at one of the representative CpG probes (cg14149672) with gene expression of PM20D1 (FPKM) for the ROSMAP cohort brain samples. An overall fit line and the fit lines stratified by the allelic doses of rs708727 are shown. **Figure S2**. Correlation of DNA methylation in blood with four brain regions (prefrontal cortex, entorhinal cortex, superior temporal gyrus and cerebellum) from 71-75 matched samples for one of the representative probes (cg11965913). The figure is taken from the Blood Brain DNA Methylation Comparison Tool (17) (https://epigenetics.essex.ac.uk/bloodbrain/index.php?probenameg=cg11965913). **Figure S3**. Methylation change as disease progresses in the AD patients, modeled by β values regressed with age at one of the representative CpG probes (cg14893161). Scatter plot is colored by the allelic doses of rs708727 where red = 0, green = 1, and blue = 2. An overall linear fit line, as well as the fit lines for each allelic dose group are also shown.

## Data Availability

The datasets supporting the conclusions of this article are available in the following repositories: ANDI: http://adni.loni.usc.edu/data-samples/access-data/. ROSMAP: https://www.synapse.org/#!Synapse:syn3219045 All the analysis results and codes are available from the authors upon request.

## Abbreviations

LOAD: Late-onset Alzheimer’s disease
QTL: Quantitative trait locus
MCI: Mild cognitive impairment
ADNI: Alzheimer’s Disease Neuroimaging Initiative
DMR: Differentially methylated region
LME: Linear mixed effects
ROSMAP: Religious Orders Study and Memory and Aging Project
WGS: Whole-genome sequencing
AMP-AD: Accelerating medicines partnership-Alzheimer’s disease
FPKM: Per kilobase of transcript per million mapped reads
SNP: Single-nucleotide polymorphism
CSF: Cerebral spinal fluid
KO: Knock out
