# Supplementary material for: Longitudinal data in peripheral blood confirm that PM20D1 is a quantitative trait locus (QTL) for Alzheimer’s disease and implicate its dynamic role in disease progression
Source: Clin Epigenetics. 2020 Dec 9;12:189. doi: 10.1186/s13148-020-00984-5 (PMC7724832; doi:10.1186/s13148-020-00984-5)

Figure S1. Correlation of methylation β values at one of the representative CpG probes (cg14149672) with gene expression of PM20D1 (FPKM) for the ROSMAP cohort brain samples. An overall fit line and the fit lines stratified by the allelic doses of rs708727 are shown.


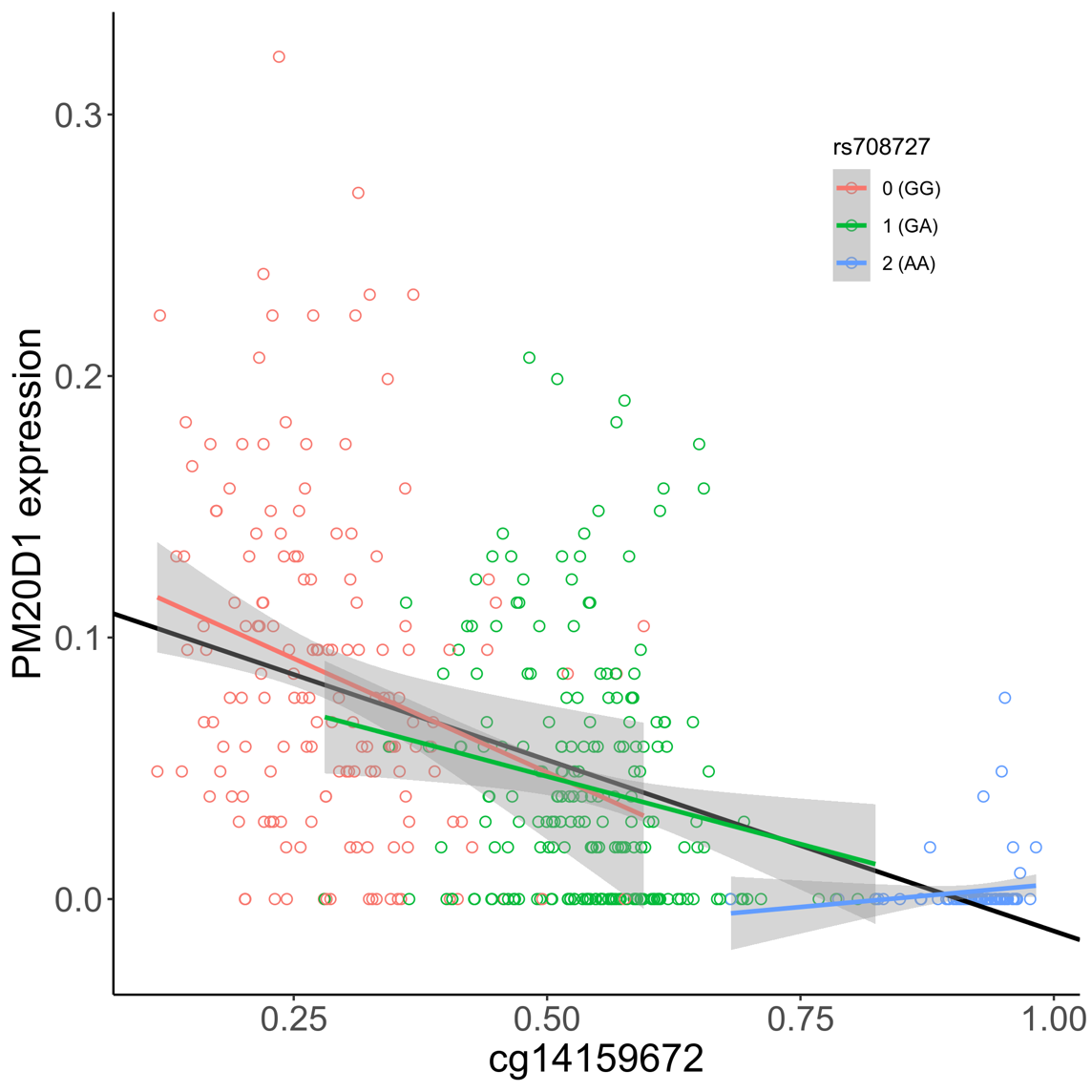


Figure S2. Correlation of DNA methylation in blood with four brain regions (prefrontal cortex, entorhinal cortex, superior temporal gyrus and cerebellum) from 71-75 matched samples for one of the representative probes (cg11965913). The figure is taken from the Blood Brain DNA Methylation Comparison Tool (17) (https://epigenetics.essex.ac.uk/bloodbrain/index.php?probenameg=cg11965913).


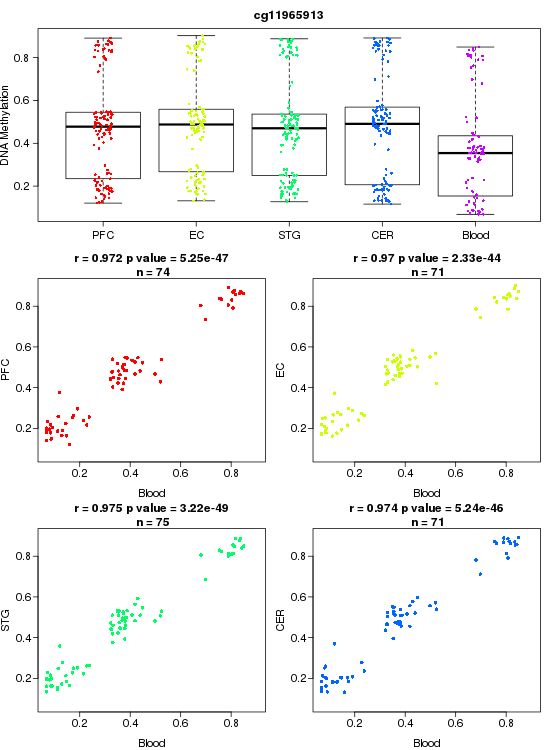


Figure S3. Methylation change as disease progresses in the AD patients, modeled by β values regressed with age at one of the representative CpG probes (cg14893161). Scatter plot is colored by the allelic doses of rs708727 where red = 0, green = 1, and blue = 2. An overall linear fit line, as well as the fit lines for each allelic dose group are also shown.


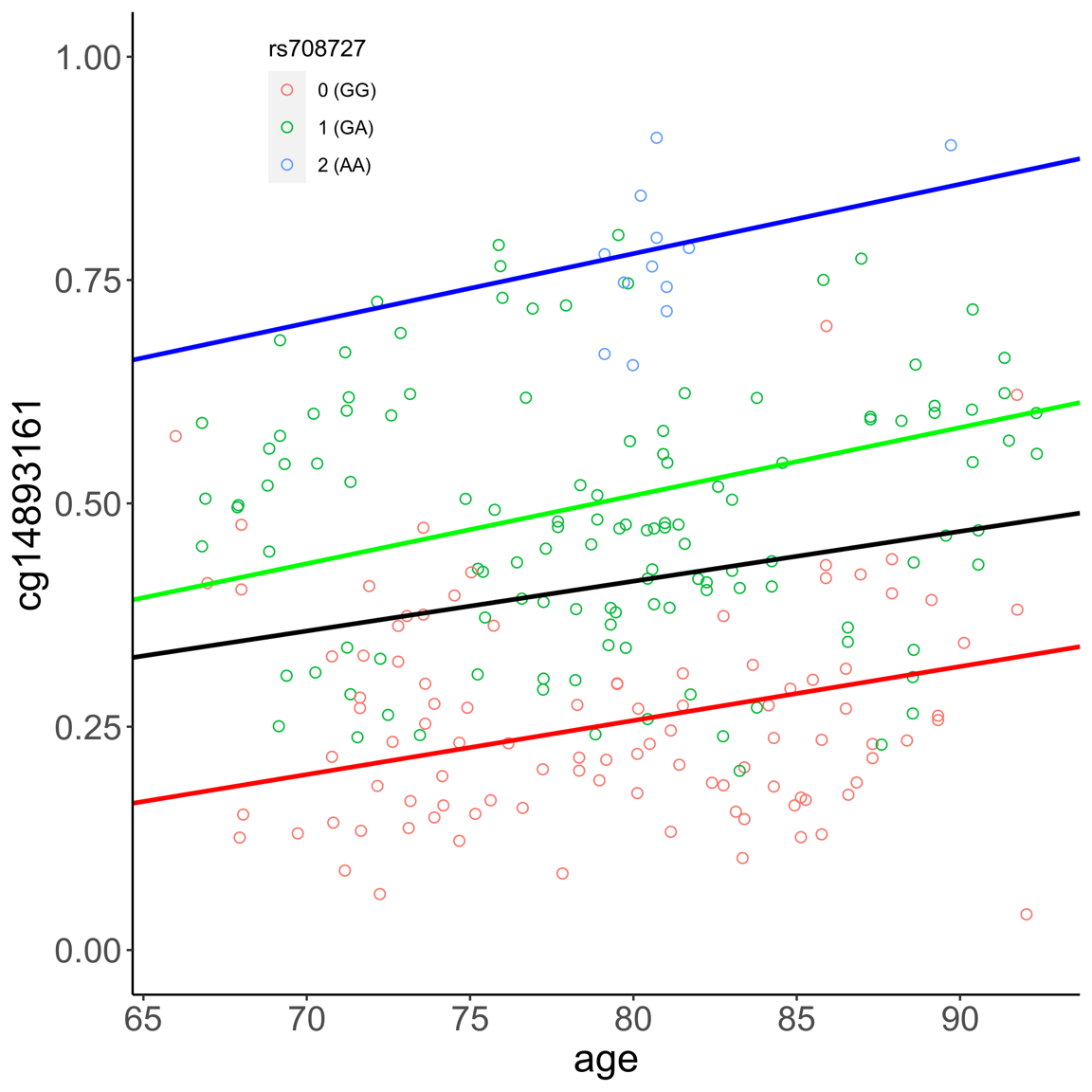

Supplement: Supplementary file 9 — Additional file 9. Figure S1. Correlation of methylation β values at one of the representative CpG probes (cg14149672) with gene expression of PM20D1 (FPKM) for the ROSMAP cohort brain samples. An overall fit line and the fit lines stratified by the allelic doses of rs708727 are shown. Figure S2. Correlation of DNA methylation in blood with four brain regions (prefrontal cortex, entorhinal cortex, superior temporal gyrus and cerebellum) from 71-75 matched samples for one of the representative probes (cg11965913). The figure is taken from the Blood Brain DNA Methylation Comparison Tool (17) (https://epigenetics.essex.ac.uk/bloodbrain/index.php?probenameg=cg11965913). Figure S3. Methylation change as disease progresses in the AD patients, modeled by β values regressed with age at one of the representative CpG probes (cg14893161). Scatter plot is colored by the allelic doses of rs708727 where red = 0, green = 1, and blue = 2. An overall linear fit line, as well as the fit lines for each allelic dose group are also shown. [file 13148_2020_984_MOESM9_ESM.docx]
